# Tolerability, Efficacy and Feasibility of Concurrent Gemcitabine and Cisplatin (CGP) Combined With Intensity Modulated Radiotherapy for Loco-Regionally Advanced Carcinoma of the Cervix

**DOI:** 10.7150/jca.40276

**Published:** 2020-02-19

**Authors:** Emmanuel Kwateng Drokow, Liu Zi, Han Qian, Lanlan Xu, Francis Foli, Hafiz Abdul Waqas Ahmed, Gloria Selorm Akpabla, Guangyin Wu, Emmanuel Bamfo Agyekum, Weihua Gao, Marie-Anne Deku, Juanjuan Song, Kai Sun

**Affiliations:** 1 Department of Radiation Oncology, Zhengzhou University People's Hospital & Henan Provincial People's Hospital 450003, China; 2 Department of Radiation Oncology, First Affiliated Hospital of Xi'an Jiaotong University, Xi'an 710061, China; 3 Department of Internal Medicine, Seventh Day Adventist Hospital, Takoradi MC 1034, Ghana; 4 Department of Haematology, Zhengzhou University People's Hospital & Henan Provincial People's Hospital 450003, China; 5 Department of Internal Medicine, Tianjin Medical University, Tianjin 300033, China; 6 Department of Pharmacy, Zhengzhou University, 450001, Zhengzhou-China.; 7 Department of Oncology, Binzhou Medical University, Yantai-Shandong 264003, China

**Keywords:** gemcitabine, cisplatin, cervical cancer, chemoradiotherapy, overall survival

## Abstract

**Background:** Gemcitabine and cisplatin combined with conventional radiotherapy in treating patients with cervical cancer, resulted in a favourable conclusion but accompanied with high toxicity. The objective of our research was to assess the tolerability, efficacy and feasibility of dual chemotherapy in addition to image-guided adaptive brachytherapy and highly conformal external beam radiation therapy.

**Methods & Materials:** From June 2011 to November 2013, 81 cervical cancer patients with FIGO stage IB2-IIIB medical records were retrospectively reviewed. All patients received whole pelvic radiotherapy (WPRT) to a total dose of 50.4 Gy/ 1.8 Gy Chemoradiotherapy prescription objectives were: concurrent gemcitabine (125 mg/m^2^) and cisplatin (30 mg/m^2^) during the 6 weeks of external beam radiation therapy (EBRT) followed by two cycles of gemcitabine (1 g/m^2^, d1, d8) and cisplatin (25 mg/m^2^ d1-d3) on the tenth week. External beam radiotherapy was followed by image-guided brachytherapy of 24 Gy/ 4 fractions. Version 4 of the common terminology criteria for adverse events (CTCAE v 4.0) was used in grading the toxicities.

**Results:** Sixty-nine patients obtained complete response (CR), six had a partial response (PR), and five patients had stable disease (SD). The disease control rate (DCR= SD and ORR) and overall response rate (ORR= PR, CR or PR) were 92.6% and 85.2% respectively. The 3-year and 5-year estimated overall survival (OS) was 75.4% and 66.3%, and the 3-year and 5-year estimated progression-free survival (PFS) were 78.2% and 65.4%. The median PFS time and OS time were 36.8 months and 45.5 months, respectively. Distance metastasis was evident in the lung (3 patients), pelvic wall (2 patients), liver (3 patients) and bone (2 patients). Six (6) had a local relapse, and two (2) patients had local relapse plus simultaneous systemic metastatic tumour.

**Conclusions:** Unlike past results, gemcitabine and cisplatin appear to be tolerable, efficient and feasible when combined with conformal radiotherapy.

## Introduction

As the second most frequently diagnosed cancer and the third major cause of death in female, cervical cancer is estimated to have a prevalence and mortality rate of 527,000 and 265,700 respectively, representing 8% of total cancer death and cases in females 1,2. Squamous cell carcinoma (80-90%) and adenocarcinoma are the common histology type seen at diagnosis 3-5. Presently, the standard treatment and management of cervical cancer are chemoradiotherapy, surgery or high dose intracavitary brachytherapy. Numerous prospective randomized control studies have demonstrated that combined cisplatin-based chemoradiotherapy reduces death rates, increases and improves disease-free survival. Furthermore, substantial evidence revealed that patients treated with cisplatin-based chemoradiotherapy have a better outcome than those who received only radiation therapy, even though it is correlated with higher side effects. The combination of this multimodal approach results in an abject 12% increase in 5-year overall survival compared to radiation alone 6.

The open phase III clinical trial conducted by Duenas et al evaluated the therapeutic advantages of chemoradiotherapy, and the results laid the basis for concomitant chemoradiotherapy (CCRT) to be the preferred therapy for loco-regionally advanced carcinoma of the cervix 7. Intensity-modulated radiotherapy (IMRT) has altered the prognosis for cervical cancer with the development of medicine and contemporary delivery techniques in radiation treatment. The inclusion of multimodal imaging, particularly in cervical cancer patients, improves systemic and or nodal tumour detection while enabling the better description of loco-regionally tumour and selection of patients. Using dose-escalated radiation therapy, it is now feasible to treat metastatic lymph nodes without serious toxicity. Notwithstanding all the progress, however, systemic disease recurrence continues to be an issue after treatment. The challenge of decreasing the recurrence of diseases is well recognized, and the future response may be to add another chemotherapeutic drug to the present accepted treatment of single-agent cisplatin. A few trials revealed promising outcomes with chemotherapies involving cisplatin and anthracycline. However, since anthracyclines are linked with serious and severe cardiac toxicity and bone-marrow suppression, less toxic non-anthracycline regimens need to be investigated.

Gonźales et al reported there was a significant reduction in toxicity when gemcitabine and cisplatin were used in advanced cervical cancer patients. The synergy between gemcitabine and cisplatin has been demonstrated by multiple preclinical and clinical trials 7. With that in mind, we chose the cisplatin and gemcitabine (GP) combination for loco-regionally advanced carcinoma of the cervix (LRACC) in our institution, since the mechanism of action of gemcitabine varies from previous agents. Furthermore, this combined regimen has demonstrated small toxic profile. Additionally, using gemcitabine plus cisplatin in treating other squamous cell carcinomas has shown promising effectiveness 8-11.

Gemcitabine is a deoxycytidine analogue and wide-spectrum antimetabolite with antineoplastic activity. The antineoplastic nature of gemcitabine has resulted in its usage in several forms of advanced cancers. Gemcitabine is a drug with single-agent action in recurrent or metastatic cervical carcinoma but has demonstrated clear radio-sensitizing characteristics in preclinical studies, even in human cervical cell lines.

The objective of our research was to evaluate the tolerability, efficacy and feasibility of dual chemotherapy using gemcitabine and cisplatin regime in addition to image-guided adaptive brachytherapy and highly conformal external beam radiation therapy in loco-regionally advanced carcinoma of the cervix.

## Method & Materials

### Patients

Histologically confirmed carcinoma of the cervix patients with FIGO stage 1B2-IIIB tumours treated with concurrent gemcitabine plus cisplatin and IMRT at the Radiation Oncology Department of Zhengzhou University People's Hospital were involved in our research. The study was approved by the institutional review and ethics board of Zhengzhou University. The pre-treatment histories, medical and follow-up records of 81 treated patients from June 2011 to November 2013 were reviewed retrospectively. The 2009 International Federation of Gynecology and Obstetrics staging system were used.

### Pre-treatment workup

All the patients included in our study underwent our institutional pre-treatment workup protocol (reviewing previous medical records**,** gynaecological and general physical examination, tumour biopsy, digital rectal examination (DRE), complete blood count (CBC), biochemical examinations, cystoscopy and rectoscopy). The eligibility criteria included:

- Histologically confirmed carcinoma of the cervix patients
- Patients without a previous history of being treated for any type of cancer
- KPS score above 70
- No evidence of distance metastasis
- Patients with normal liver functions

The exclusion criteria included patients with evidence of distance metastasis, benign tumours, and cervical sarcoma patients.

### Radiotherapy Planning

Imported into the pinnacle treatment planning system (TPS) were images obtained by diagnostic 18FDG-PET/CT, CT and MRI. The RTOG guidelines were used in contouring. Based on all accessible medical and imaging information, the gross tumour volume (GTVc) was described as the visible macroscopic tumour. The clinical target volume (CTV) involved the regional lymph nodes and primary tumour sites. The primary clinical target volume encompassed gross tumour volume, parametrium, cervix, uterus and upper one-third of the vagina. In instances, where there was involvement of the vagina, the clinical target volume was extended 20 mm into the tumour's vagina caudal. The planning target volume (PTV) was generated using an isotropic expansion with the internal target volume (motion of uterus, cervix and surrounding organs) taken into account. The planning target volume was expanded 1cm laterally and 1.5 cm in the anterior-dorsal direction. Correction of the planning volume was done when necessary 12-14.

The PTV was prescribed to a total dose of 50.4 Gy. Tumour response assessment using magnetic resonance imaging was done after patients had received 45Gy of the total dose. In situations where the rest of the tumour was bigger than four cm in diameter, the PTV received an additional 9 Gy boost while tumours less than four cm after evaluation also received an additional 5.4 Gy boost. The patient received 1.8 Gy per fraction. The organs at risk (OAR) dose constraints were: 50% of bladder volume must not receive above 50Gy, 35% of the intestines must not receive dose above 35Gy, 60% of rectal volume must not receive dose above 50Gy, and 10% of the femoral heads must not receive above 50Gy.

All patients received image-guided adaptive brachytherapy (IGABT) dose of 6 Gy in four-fractions (24 Gy) and the total equivalent dose in 2 Gy fraction (EQD_2_) is 32Gy. The vagina was adequately packed with gauze to keep the rectum and bladder away from receiving a high dose of radiation. Images set obtained by CT- MRI scan was transferred to the Oncentra planning system. microSelectron (Iridium-192) was used during the treatment.

### Chemotherapy

Intravenously infused 30 mg/m^2^ of cisplatin were received by all patients over half an hour and instantly followed by intravenously infused 125 mg/m^2^ of gemcitabine about half an hour for 6 weeks thus once per week combined with radiation therapy for 6 weeks from Monday - Friday. Both drugs were given 1 to 2 hours prior to radiation therapy. Patients received adaptive image-guided brachytherapy (IGABT) on the 7th week immediately after completing chemoradiotherapy plan which, was followed by 14 days of rest. Patients subsequently received two successive 3 weeks' cycle of adjuvant chemotherapy with 1000 mg / m^2^ of gemcitabine on days 1 and 8 and 25 mg / m^2^ of cisplatin on day 1-3.

### Treatment Evaluation

Multiple parameters were controlled in accordance with the institutional standards of Zhengzhou university people's hospital and were required to meet the standard safety criteria before the commence of every chemotherapy cycle. Haematology and chemistry results were obtained weekly before starting every cycle of chemotherapy. Chemoradiotherapy was paused when the leucocytes were < 2000/mm^3^, thrombocytes <100, 000/ mm^3^, or neurotoxicity below grade 3 per CTCAE v4. Blood transfusion was given to patients with haemoglobin (Hb) level of less than 80 g/l. Non-haematological and haematological side effects due to chemotherapy were recorded based on the Common Terminology Criteria for Adverse Effects Version 4.0 The Response Evaluation Criteria in Solid Tumours (RECIST v1.1) was used in evaluating the response of the tumour to the treatment.

### Statistical Method

Statistical assessment was conducted using SPSS Version 23.0. The median PFS and median OS were evaluated with the Kaplan - Meier curve. All the data recorded was entered into the excel file and evaluated in proportions and percentages whenever necessary.

## Results

### Patients Clinical Characteristics

The clinical features of the 81 patients involved in our analysis are presented in Table 1. The median age was 45 (Range: 25-60). Sixty-six patients had the squamous cell carcinoma histological subtype, and fifteen (18.5%) had adenocarcinoma subtype. The cervix tumour varied from about 3.5-8.1 cm at their greatest dimension and the median size was 4.5 cm. Symptoms at diagnosis were vaginal bleeding, vaginal discharge and dyspareunia. Seven patients (8.6%) were stage IB2, 10 patients (12.3%), 27 patients (33.3%), 13 patients (16%) and 24 patients (29.6%) were stage IIA, IIB, IIIA and IIIB respectively based on the 2009 FIGO guidelines for staging cervical cancer. Thirty-five (35) patients (43.2%) had positive para-aortic lymph node.

### Survival Outcomes and treatment efficacy

**Figure 1:**
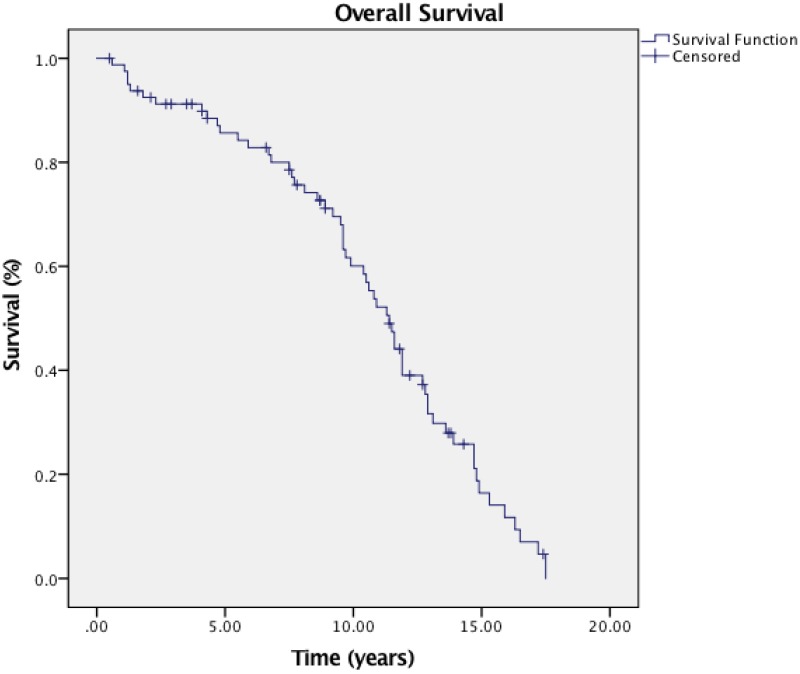
Overall survival curve of patients.

**Figure 2:**
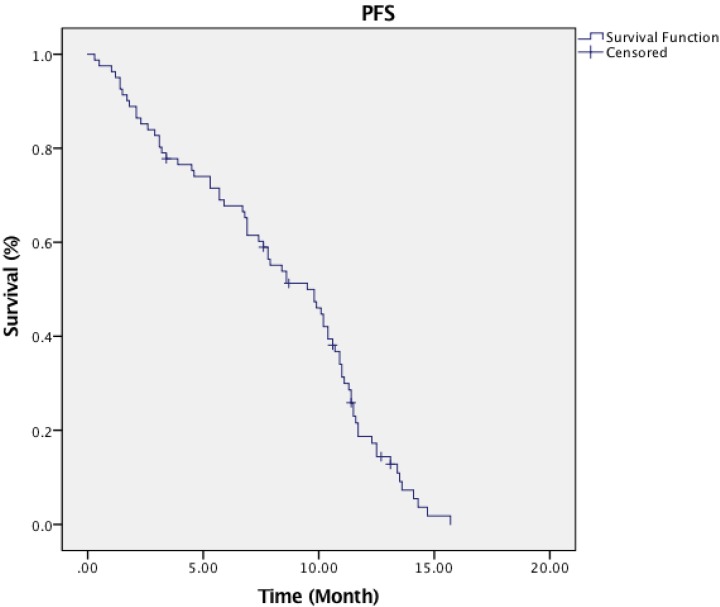
Progression-free survival curve of patients.

Short-term therapy responses were assessed three months after concurrent chemoradiotherapy and IGABT. Sixty-nine patients obtained complete response (CR), six had a partial response (PR), and five patients had stable disease (SD). The disease control rate (DCR= SD and ORR) and overall response rate (ORR= PR, CR or PR) were 92.6% and 85.2% respectively. The 3-year and 5-year estimated OS was 75.4% and 66.3%, and the 3-year and 5-year estimated PFS were 78.2% and 65.4% **(Figure 1 & 2).** The median OS time and PFS time were 45.5 months and 36.8 months, respectively. Distance metastasis was evident in the lung (3 patients), pelvic wall (2 patients), liver (3 patients) and bone (2 patients). Six (6) had a local relapse, and two (2) patients had local relapse plus simultaneous systemic metastatic tumour.

### Early and Late Clinical Toxicity

The toxicities of concurrent chemoradiotherapy were graded by CTCAE v4. No interruption was observed during the treatment. The treatment was completed by all patients and toxicity was well tolerated. Nausea was recorded in 9 (8.6%) patients, 4 patients were grade 1 (4.9%), 3 were grade 2, and 2 patients were grade 3. Eight patients and 5 patients had grade 1 and grade 2 diarrhoea, respectively. Ten (10) patients had anorexia, and 6 patients had vomiting. (4 were grade 1, and 2 were grade 2). Thrombocytopenia was observed in 8 patients, and 10 patients had anaemia. Seven patients had grade 1 neutropenia. Late genitourinary and gastrointestinal toxicity was noticed in 13 (16.0%) patients. Six (7.4%) had grade 1upper gastrointestinal toxicities, 5 patients (6.2%) had grade 1 cystitis, and one patient had grade 2(1.2%) cystitis. Grade 1 dryness of the vagina was evident in 4 (4.9%) patients, two had grade 1 (2.5%) shortening of vagina, and three (3.7%) patients had grade 2 vaginal stenosis. Table 2 shows the summary of early and late toxicities.

## Discussion

Radiotherapy alone has unsatisfying outcomes in the management and treatment of locoregionally advanced carcinoma of the cervix. Several studies have been designed and undertaken over the last decade with the objective of improving treatment result. In a meta-analysis performed by Vale C et al on individual patient information, the combined findings of these crucial studies were assessed 10. ⠀The inclusion of chemotherapy decreased both distant and local recurrence and contributed to about a 6% increase in 5-year survival when compared to only radiotherapy 15. Chemoradiotherapy with cisplatin was recognized as the standard care of treatment after the National Cancer Institute notice in 1999. Cisplatin is well tolerated on a weekly basis and demonstrates better outcomes in research compared to radiation therapy alone. Nevertheless, the cure rate remains low, and the 5-year response rate is about 60% 16.

In concurrent chemoradiotherapy protocols, non-platinum compounds alone were rarely evaluated and were usually overlooked in the literature. In a few studies, 5-FU (fluorouracil) and cisplatin were tested, but the problem was toxicity, and the dimension of frequent treatment gaps was also highlighted. Four out of the five studies in which cisplatin was accepted to be the standard treatment had 5-FU combined with cisplatin 16-17. Equally, gemcitabine remains another underused chemotherapeutic agent in concomitant chemoradiotherapy setting of cervical cancers. Compared to the conventional concurrent cisplatin routine in cervical cancer, we do not have much information on its effectiveness due to the absence of studies.

Gemcitabine is part of the group of antimetabolites (2', 2'-di-fluorodeoxycytidine) that inhibits the synthesis of DNA and ultimately causes programmed cell death. Gemcitabine has demonstrated effectiveness against a multitude of tumours, particularly lung and pancreatic cancer. It was also evaluated in gastrointestinal (GI) cancers, breast, urinary bladder, ovary and cancer of the cervix 18. Gemcitabine was originally tested for moderate activity in recurrent and metastatic cervical cancers. The findings were comparatively small when used alone in post-radiation or recurrence residual tumours. Gemcitabine is rarely used alone in treating cervix carcinoma in a residual/recurrence setting owing to less than anticipated outcomes 16,18.

Studies *in vivo* and *in vitro* have shown that gemcitabine is a powerful radiosensitizer that dramatically increases the outcomes when coupled with radiotherapy 17,19. It also demonstrates synergistic action with cisplatin, the likely cause being DNA cisplatin adducts repair suppression by gemcitabine 19.

Burnett et al. reported a total response rate of 41% with a reasonable toxicity effect 20. The neoadjuvant application of gemcitabine plus cisplatin chemotherapy was investigated by González et al 21-22. They reported that the most prevalent haematological toxicity was grade 3 granulocytopenia in 13.8% of the patients and grade 4 granulocytopenia in 3.4% of the patients. The further evaluation also demonstrated that the neoadjuvant strategy is at least as efficient as standard concomitant chemoradiotherapy involving cisplatin.

A phase I and II trial conducted by Zarba et al investigated weekly gemcitabine and cisplatin in thirty-six patients. They discovered that a peak acceptable dose of 150 mg / m^2^ is the peak tolerability dose for gemcitabine when used weekly during concurrent chemoradiotherapy 23. In addition, Umanzor et al. reported well-tolerated toxicity in 23 patients treated with concurrent gemcitabine plus cisplatin. In the same study, grade 3 neutropenia was evident in one patient. Secondly, no haematological toxicity of grade 4 and no unusual toxicity of late radiation was observed 24. A pivotal study conducted by González et al endorsed those early studies. The researchers compared concurrent chemoradiotherapy involving cisplatin and gemcitabine followed by adjuvant cisplatin and gemcitabine in locally advanced carcinoma of the cervix. Their results showed an improved 3-year OS [HR:0.68, 95% CI: 0.49-0.95, p-value=0.022]. Similar improvement was achieved in 3-year PFS [74.4% vs 65.0%, p-value=0.029] and lower distant metastasis rate [8.1% vs 16.4%, p-value=0.005]. Nevertheless, grade 3 and 4 toxicity was coupled with this success [86.5% vs. 46.3%, p-value = 0.001] 7.

Compared to prior studies, our research has several unique methodological and patient characteristics. First, we used highly conformal radiotherapy in treating our patients while conventional external beam radiotherapy was used in previous studies. This is the possible reason for the lower rate of haematological toxicity achieved in our studies. In addition, IMRT has been shown to be superior to three-dimensional (3D) and two-dimensional (2D) methods, both in terms of reducing toxicity incidence and bone-marrow sparing 25-26.

The findings of our study are similar to those reported in the literature. Loco-regional control is superior to past studies that used the gemcitabine plus cisplatin regimen. This can be ascribed to the use of intrauterine adaptive brachytherapy other than chemotherapy inclusion. Local failure was a problem in González et al study, though there was no statistical difference when the rate of local failure was considered (16.4% vs. 11.2%, p= 0.097). Nevertheless, tumour control is challenging since systemic metastasis is reported in about 40% of patients 7.

Modern radiation therapy technique has resulted in outstanding and excellent local control rates. Regardless of tumour size the 3-year local control rate goes beyond 95% and approaches approximately 100% if the equivalent total dose (EQD2) to the volume of the "high-risk tumour'' is higher than 87 Gy 27. These observations lead to the hypothesis that the combinations of chemotherapy during radiotherapy have an important impact. Moreover, even without visible local tumour at the moment of chemoradiotherapy, the microscopic tumour could still be harboured in the local lymph nodes, and the macroscopic tumour may likewise be available and stay unnoticed by radiological examinations like MRI or computed tomography 28-29. It is likely that giving chemotherapy during external beam radiotherapy may contribute to the effective eradication of tumours in the lymph nodes.

Small sample size, short-follow up, and the retrospective nature of the study are some of the limitations in our study. Notwithstanding these restrictions, we believe our research offers a distinctive perspective into new therapeutic options for patients with locoregionally advanced carcinoma of the cervix. The data presented can serve as a foundational guide for future randomized controlled trials.

## Figures and Tables

**Table 1:** Patients clinical characteristics.

Characteristics	NO. Patients	(n %)
**Age**		
< 55	63	77.8%
≥ 55	18	22.2%
**Histology type**		
SCC	66	81.5%
Adenocarcinoma	15	18.5%
**Stage**		
IB2	7	8.6%
IIA	10	12.3%
IIB	27	33.3%
IIIA	13	16.0%
IIIB	24	29.6%
**Grade**		
1	6	7.4%
2	54	66.7%
3	21	25.9%
**Tumour Size**		
< 4 cm	23	28.4%
≥ 4 cm	58	71.6%
**LNM**		
1	26	32.1%
2	21	25.9%
3& above	34	42.0%
**Para-aortic LNM**		
Yes	35	43.2%
No	46	56.8%

**Table 2:** Early and late toxicities during treatment.

Toxicity	Grade			
	1	2	3	4
Anaemia	8 (9.9%)	2 (2.5%)	0	0
Neutropenia	6 (7.4%)	1 (1.2%)	0	0
Thrombocytopenia	5 (6.2%)	3 (1.4%)	0	0
Nausea	4 (4.9%)	3 (7.1%)	2 (2.5%)	0
Vomiting	4 (4.9%)	2 (2.5%)	0	0
Diarrhea	8 (9.9%)	5 (6.2%)	0	0
Anorexia	8 (9.9%)	2 (2.5%)	0	0
Late gastrointestinal	7 (8.6%)	0	0	0
Late genitourinary	5 (6.2%)	1 (1.2%)	0	0

## Abbreviations

CCRT: concomitant chemoradiotherapy
LRACC: loco-regionally advanced carcinoma of the cervix
PTV: Planning target volume
CTV: Clinically target volume
RTOG: Radiation Therapy Oncology Group
Gy: Gray
